# 

**Published:** 1933

**Authors:** 


### THE BRISTOL MEDICO-CHIRURGICAL JOURNAL

### COMBINED INDEX

*for*

Volumes 1 to 50

1883 — 1933

INCLUSIVE

---

"Scire est nescire, nisi id me

Scire alius sciret"

---

### CONTENTS

| | PAGE |
|---|---|
| Foreword | 3 |
| Editorial Staff | 4 |
| Presidents and Presidential Addresses | 5 |
| The Long Fox Memorial Lectures | 9 |
| Index to Authors | 11 |
| Index to Subjects | 33 |
| Obituaries | 56 |

ARMY MEDICAL LIBRARY
312169
WASHINGTON, D.C.

BRISTOL: J. W. ARROWSMITH LTD.

LONDON: J. W. ARROWSMITH (LONDON) LTD.

